# The Landscape of Interactions between Hypoxia-Inducible Factors and Reactive Oxygen Species in the Gastrointestinal Tract

**DOI:** 10.1155/2021/8893663

**Published:** 2021-01-21

**Authors:** Yirui Shao, Kexing Wang, Xia Xiong, Hongnan Liu, Jian Zhou, Lijun Zou, Ming Qi, Gang Liu, Ruilin Huang, Zhiliang Tan, Yulong Yin

**Affiliations:** ^1^Hunan Province Key Laboratory of Animal Nutritional Physiology and Metabolic Process, Key Laboratory of Agro-ecological Processes in Subtropical Region, National Engineering Laboratory for Pollution Control and Waste Utilization in Livestock and Poultry Production, Institute of Subtropical Agriculture, Chinese Academy of Sciences, Changsha, 410125 Hunan, China; ^2^University of Chinese Academy of Sciences, Beijing 100008, China; ^3^Laboratory of Animal Nutrition and Human Health, College of Life Sciences, Hunan Normal University, Changsha 410081, China; ^4^Laboratory of Basic Biology, Hunan First Normal University, Changsha 410205, China; ^5^College of Bioscience and Biotechnology, Hunan Agricultural University, Changsha 410128, China

## Abstract

The gastrointestinal tract (GT) is the major organ involved in digestion, absorption, and immunity, which is prone to oxidative destruction by high levels of reactive oxygen species (ROS) from luminal oxidants, such as food, drugs, and pathogens. Excessive ROS will lead to oxidative stresses and disrupt essential biomolecules, which also act as cellular signaling molecules in response to growth factors, hormones, and oxygen tension changes. Hypoxia-inducible factors (HIFs) are critical regulators mediating responses to cellular oxygen tension changes, which are also involved in energy metabolism, immunity, renewal, and microbial homeostasis in the GT. This review discusses interactions between HIF (mainly HIF-1*α*) and ROS and relevant diseases in the GT combined with our lab's work. It might help to develop new therapies for gastrointestinal diseases associated with ROS and HIF-1*α*.

## 1. Introduction

The gastrointestinal tract (GT) is the major place of nutrient digestion and absorption, and it is prone to oxidative destruction by highly reactive oxygen species (ROS) [1, 2]. Reactive oxygen species are byproducts of normal cellular metabolism. They usually contain an unstable number of electrons, and they make them extraordinarily reactive. Excessive ROS do harm to essential biomolecules, including nucleic acids, proteins, and lipids. Accumulated ROS will lead to oxidative stresses, which contribute to various diseases in the GT [3]. However, ROS also act as important signaling molecules in response to growth factors, hormones, and oxygen tension changes [4, 5]. Hypoxia-inducible factors (HIFs) are indispensable transcription factors in response to low oxygen [6]. HIFs have drawn lots of attention from researchers for their role involved in gastrointestinal energy metabolism, immunity, renewal, and microbial homeostasis. Intriguingly, there are close relationships between ROS and HIFs. This review is aimed at summarizing interactions between HIFs and ROS in the GT to help develop new treatments for gastrointestinal diseases induced by ROS and HIFs.

## 2. The Role of ROS in the GT

ROS include superoxide (O_2_^·–^), hydroxyl radicals (OH^·^), hydrogen peroxide (H_2_O_2_), singlet oxygen (^1^O_2_), hypochlorous acid (HOCl), chloramines (RNHCl), and ozone (O_3_) [7]. Gastrointestinal ROS are usually classified as endogenous and exogenous ROS. As has been reviewed by Bhattacharyya et al., endogenous ROS mainly comes from mitochondrial electron transport chain (mETC), NADPH oxidase (NOX), cyclooxygenase (COX), myeloperoxidase (MPO), lipoxygenases (LOXs), xanthine oxidase (XO), and transition metals; correspondingly, food, drink, xenobiotics, cigarettes, and radiation lead to exogenous ROS in the GT [8].

In addition, toxins and environmental stresses can also contribute to exogenous ROS in the GT. Reportedly, aflatoxin B_1_ and aflatoxin M_1_ treatment inhibited cell viability, enhanced LDH release, and led to DNA damage in Caco-2 cells, which was related to elevated ROS [9]. Patulin also induced endoplasmic reticulum stress and mitochondrial apoptosis in human intestinal cells in the ROS-dependent way [10]. Our lab's work also showed that deoxynivalenol treatment significantly upregulated the MDA levels and downregulated the total antioxidant capacity, which further led to reduced villus height and increased lymphocytes in the piglet ileum and jejunum [11]. The environment is a major source of ROS in the GT. For instance, heat stress led to elevated ROS and MDA levels and reduced antioxidase activity, accounting for increased apoptosis and intestinal permeability in the rat small intestine [12]. In addition, noise also contributes to oxidative stress. Noise-treated rats suffered mast cell degranulation, damages to the endothelial cell membrane, and increased eosinophils in the lamina propria of villi in the intestine, which might be caused by excessive ROS [13]. Additionally, transport stress contributed to oxidative stresses in the intestine, including increased MDA, endotoxin, NOX1, and lactic dehydrogenase (LDH) levels and decreased expression of SOD and tight junction proteins [14]. Our lab's work also showed that weaning stress decreased antioxidant enzyme levels and affected the expression of genes involved in ROS generation in piglets [15]. As mentioned above, multiple variables can affect the generation of ROS and, eventually, lead to oxidative stress-induced diseases in the GT.

Excessive ROS can do harm to DNA, proteins, and lipids. Further, we found that ROS significantly inhibited the proliferation rate, mitochondrial respiration, and antioxidative capacity and contributed to cell apoptosis in IPEC-J2 cells [16]. However, ROS also act as signaling molecules and contribute to defense systems in the body. ROS act as the activator of Ca^2+^ permeable cationic channels formed by transient receptor potential melastatin 2 protein in immune cells [17]. Tyrosine-protein kinase Lyn is a member of the Src family of protein tyrosine kinase, which is involved in multiple cellular signaling transduction [18]. ROS are critical for intestinal epithelial cell activities. NOX-derived O_2_^·–^ has been reported to augment host defense in colon epithelial cells [19]. Additionally, NOX-derived ROS plays a key role in modulations of the actin cytoskeleton, monolayer permeability, cell migration, cell proliferation, and focal adhesion kinase phosphorylation [20]. Further, ROS participate in the regulation of bacteria to the host. VvpE is an elastase encoded by an opportunistic gram-negative pathogen *Vibrio vulnificus* [21]. And lipid raft-mediated ROS signaling is essential for the inhibition of mucin 2 expression by VvpE in the intestinal epithelial cells [22]. Further, our work also showed that the low dosage of H_2_O_2_ might play a feedback regulatory role against oxidative injury via upregulating the expression of *UCP2* and mitochondrial proton leak in IPEC-J2 cells [23]. ROS act as signaling molecules in the modulation of various signaling pathways, leading us to investigate whether ROS participate in the regulation of HIF-1*α*.

## 3. The Role of HIFs in the GT

HIF-1 was first discovered by Semenza and Wang when they studied the transcription of the hypoxia-inducible human erythropoietin gene [24]. HIFs are heterodimers consisting of hypoxia-inducible *α*-subunit (HIF-1*α*, HIF-2*α*, and HIF-3*α*) and *β*-subunit (aryl hydrocarbon receptor nuclear translocator (ARNT)/HIF-1*β* and ARNT2) [25]. Among them, HIF-1*α* is the most ubiquitously expressed [26].

The degradation of HIF-*α* is mainly regulated on the posttranslational level [27]. HIF-*α* is continuously synthesized, and it is rapidly degraded by proteasomal pathways under normoxia. However, under hypoxia, the degradation of HIF-*α* is hindered. As reviewed by Strowitzki et al., the degradation is mainly regulated by HIF prolyl 4-hydroxylases (PHDs): PHD1, PHD2, and PHD3; two prolyl residues in the oxygen-dependent degradation domain of HIF-*α*-subunits are hydroxylated by PHDs, contributing to the ubiquitination mediated by von Hippel-Lindau (VHL) ubiquitin ligase; factor-inhibiting HIF (FIH) can hydroxylate an asparagine residue in the C-terminal transactivation domain of HIF-1*α*; under hypoxic conditions, the hydroxylation of HIF-*α* by PHD and FIH is retarded; accumulated HIF-*α* forms complexes with HIF-*β* and translocates to the nucleus to initiate transcription of target genes [28].

**Figure 1:**
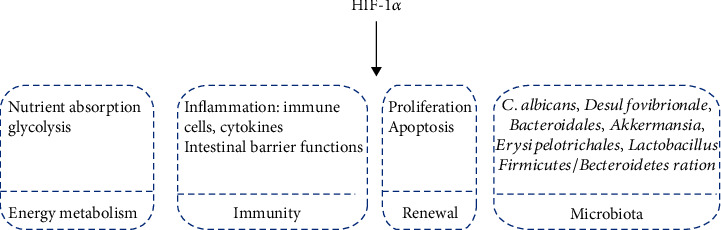
The role of HIF-1*α* in the gastrointestinal tract. HIF-1*α* is involved in gastrointestinal energy metabolism, immunity, renewal, and microbiota.

The physiological gastrointestinal mucosa has a uniquely steep oxygen gradient that the vascularized subepithelial mucosa is rich in oxygen while the luminal epithelium is hypoxic [29]. Moreover, under pathological states such as inflammation, the GT often has reduced oxygen levels [30, 31]. As the central sensor of hypoxia, HIF-1*α* governs the transcription of numerous genes, which also act as a double-edged sword in energy metabolism, immunity, renewal, and microbial homeostasis of the GT (Figure 1).

HIF-1*α* takes part in gastrointestinal energy metabolism, including glycolysis and nutrient absorption (glucose, lipid, and glutamine) [32, 33]. It was also an essential regulator in gastrointestinal immunity. The knockout of HIF-1*α* led to a significant decrease of CD8*αα*^+^ and TCR*γδ*^+^ population in intestinal epithelial cells of mice [34]. Further, activation of HIF-1*α* attenuated *C*. *difficile*-induced colitis in mice [35]. In line with this, suppression of the HIF-1*α*/COX-2 pathway contributed to the releases of inflammatory cytokines induced by NF-*κ*B p65 in the porcine ileum [36]. However, there were also studies that suggest that HIF-1*α* contributes to pathological progress in gastrointestinal diseases. As has been reported, Jian-Pi Qing-Chang treatment attenuated the intestinal epithelial permeability and inflammation by inhibiting NF-*κ*B/HIF-1*α* pathways in colitis mice [37]. Further, the interferon-gamma (IFN-*γ*) induced intestinal epithelial permeability and disruption of the intestinal tight junction by activating HIF-1*α* [38]. Similarly, berberine treatment can suppress the activation of HIF-1*α*, thus alleviating IFN-*γ*- and TNF-*α*-induced intestinal epithelial barrier dysfunction [39].

Further, HIF-1*α* played a key role in the renewal of the GT. A recent study reported that downregulated HIF-1*α* inhibited the proliferation of gastric cancer cells [40, 41]. Moreover, the transcription factor Krüppel-like factor 2 overexpression inhibited proliferation and promoted apoptosis by suppressing the expression of Notch-1 via inhibition of HIF-1*α* in colorectal cancer cells [42]. In line with this, the 4-(2-phenylpyridin-4-yl)pyrazoles exerted antiproliferation and apoptosis-inducing effect by inhibiting the activation of HIF-1*α* in HCT116 cells [43]. However, the evodiamine treatment suppressed proliferation and induced apoptosis by upregulating bone morphogenetic protein 9 (BMP9), which could activate p53 via the upregulation of the HIF-1*α* in HCT116 cells [44].

There is a strong correlation between HIF-1*α* and intestinal microbiota. The activation of HIF-1*α* significantly suppressed the colonization of *C*. *albicans* and mortality from invasive disease by enhancing the expression of CRAMP in the mouse colon [45]. Further, there was a significant decrease in the *Firmicutes*/*Bacteroidetes* ratio and *Lactobacillus* abundance, as well as an increased abundance of *Akkermansia* in intestinal epithelial-specific HIF-1*α* knockout mice [46]. In line with this, it was reported that the abundance of *Erysipelotrichales* and *Lactobacillales* increased while the abundance of *Bacteroidales* and *Desulfovibrionale* decreased in intestinal epithelial-specific HIF-1*α* knockout mice [34].

HIF-1 has been reported to be regulated by other stimuli in addition to oxygen, including hormones such as insulin, growth factors such as platelet-derived growth factor, transforming growth factor-beta, and insulin-like growth factor, and vasoactive peptides such as angiotensin-2 [47]. Apart from these nonhypoxic stimuli, a few studies suggest that ROS also participate in the regulation of HIF-1*α* in the GT.

## 4. Mechanism of HIF-1*α* Regulation by ROS in the GT

### 4.1. ROS Regulate the Expression of HIF-1*α*

As important cellular signal molecules, ROS are implicated in numerous signal MAPK, PI3K/Akt/mTOR, and NF-*κ*B pathways, which further regulate the expression of HIF-1*α*. Under hypoxia, ROS activated ERK1/2 and further enhanced the HIF-1*α* transcriptional activity, leading to the photodynamic therapy-resistant phenotype in colorectal spheroids [48]. Further, both endogenous and exogenous H_2_O_2_ could upregulate the expression of HIF-1*α* via activating PI3K/Akt/mTOR pathways in human colorectal carcinoma cells [49, 50]. Accordantly, H_2_O_2_ treatment activated Akt and ERK and subsequently increased the expression of HIF-1*α* as well as its target genes in gastric mucosal epithelial cells, which could be reversed by ROS scavengers [51]. In line with this, the expression of HIF-1*α* was mediated in an ERK-dependent way under hypoxia in H_2_O_2_-treated gastric cancer cells [52].

Moreover, ROS can activate I*κ*B kinase (IKK), which contributes to the degradation of I*κ*B and the release of NF-*κ*B proteins [53, 54]. Activated NF-*κ*B played a key role in the transcription of HIF-1*α*. As p50 and p65 directly bounded to the promotor of HIF-1*α*, overexpression of p50 and p65 enhanced expression of HIF-1*α* mRNA while elevated expression of I*κ*B had a reverse effect under hypoxia [55]. Consistently, treating human intestinal epithelial cells with the IKK2 inhibitor led to diminished expression of HIF-1*α* protein, suggesting NF-*κ*B acted as upstream of HIF-1*α* [56]. In line with this, it is reported that ROS upregulated the expression of HIF-1*α* via activating the NF-*κ*B pathway in gastric cancer cells, which could be attenuated by antioxidants [57].

### 4.2. ROS Regulate the Stability and Activity of HIF-1*α*

Under normoxia, HIF-1*α* is rapidly degraded via hydroxylation by PHDs, binding to VHL, ubiquitylation, and proteasomal degradation. During hypoxia, there are intracellular generations of ROS and NO [58], and NO can react with ROS to form RNS [59], which can modulate the posttranslation of proteins by *S*-nitrosation [60]. As has been reported, RNS, formed by endogenous ROS and NO, induced the *S*-nitrosation of PHD2, contributing to the elevated stabilization of HIF-1*α* in HCT116 cells [58]. Besides, an earlier study reported that brusatol treatment diminished the production of mitochondrial ROS, leading to the activation of PHDs and subsequent degradation of HIF-1*α* in colorectal cancer cells [61].

VHL is the major component of E3 ubiquitin ligases, which regulates the ubiquitylation and consequent proteasomal degradation of HIF-*α*. An earlier study reported that indomethacin treatment led to elevated expression of VHL through oxidative stress in IEC6 cells [62]. Further, increased VHL contributed to the degradation of HIF-1*α*, which can be reversed by the MnSOD mimetic [62].

The bHLH- (basic helix-loop-helix-) PAS (Per/ARNT/Sim) domains of HIF mediate the generation of the heterodimer and DNA binding [63]. The activity of HIF-1*α* will be suppressed when its association with ARNT is retarded [64]. An early study reported that curcumin treatment degraded ARNT via the ubiquitin-proteasome system in the ROS-dependent way, leading to the inhibition of HIF-1 in MKN28 cells [65].

## 5. HIF-1*α* Regulated by ROS Plays a Role in Gastrointestinal Diseases

**Figure 2:**
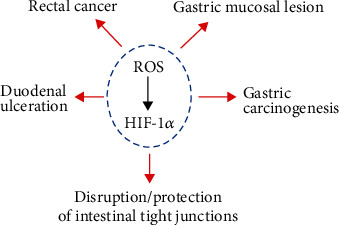
HIF-1*α* regulated by ROS plays a role in gastrointestinal diseases. HIF-1*α* regulated by ROS contributes to rectal cancer, gastric carcinogenesis, gastric mucosal lesions, duodenal ulceration, and disruption/protection of intestinal tight junctions.

Different sources of ROS participate in the modulation of HIF-1*α* and further play a role in the pathologic progress of various gastrointestinal diseases (Figure 2). There is evidence that cigarette smoke exposure led to increased ROS, contributing to the disruption of intestinal tight junctions by upregulating HIF-1*α* expression in the rat small intestine [66]. Further, elevated ROS mediated by NOX2 upregulated the expression of HIF-1*α* in the small intestine and rectal cancer cells, contributing to rectal cancer cell proliferation [67]. ROS derived from *Helicobacter pylori*-infected gastric epithelial cells mediated the expression of HIF-1*α* and its target gene vascular endothelial growth factor (VEGF), which contributed to gastric carcinogenesis [68]. Further, HIF-1*α* may lead to high-altitude polycythemia- (HAPC-) induced gastric mucosal lesions (increased apoptosis, microvessel density, and swollen mitochondria) in a ROS-mediated signaling pathway [69]. Moreover, limb ischemia reperfusion-induced ROS contribute to the proliferation of gastric epithelial cells and vascular endothelial cells of gastric tissue by upregulating HIF-1*α* expression [70]. However, *Clostridium difficile* toxin-mediated ROS play a key role in the stabilization of HIF-1*α*, leading to the innate protection of colon epithelial barrier function, which suggested that HIF-1*α* played a dual role in gastrointestinal diseases [71].

Mitochondria are the major source of intracellular ROS. However, there is a debate about whether mitochondrial ROS can regulate the expression of HIF-1*α*. It is reported that brusatol treatment downregulated mitochondrial ROS levels, leading to diminished HIF-1*α* protein levels and cell death in colorectal cancer under hypoxia [61]. In contrast, treating SNU-638 cells with the mitochondrial electron transport system inhibitor (rotenone, amobarbital, antimycin A, and KCN) did not affect the expression of HIF-1*α* protein [72]. The difference may be due to different cell types.

Moreover, the glutathione system is critical for the regulation of nonhypoxic HIF-1*α* in gastrointestinal diseases. *N*-Acetylcysteine, a thiol-containing antioxidant, is a precursor of reduced glutathione and a direct ROS scavenger [73]. Under hypoxia, *N*-acetylcysteine treatment can significantly decrease the expression of HIF-1*α* by diminishing ROS, suppressing the survival and invasion ability of gastric cancer cells under hypoxia [74]. Cysteamine, the reduced form of cystamine, contributes to the generation of glutathione, one of the most important antioxidants [75]. However, cysteamine treatment has been shown to decrease the activities of SOD and GSH-PX, leading to increased ROS in the rat duodenum [76], and augmented the HIF-1*α* expression in the early stage of duodenal ulceration [77].

Further, a variety of antioxidants can modulate HIF-1*α* expression via affecting ROS levels in the GT. For example, vitamin E supplementation diminished HIF-1*α* protein expression, protecting from intestinal injury induced by hypoxia in the rat ileum [78]. Quercetin and its metabolite isorhamnetin possess antioxidation effects in different tissues [79]. Under hypoxia, quercetin treatment reduced the activity of HIF-1*α* and enhanced apoptosis in colon cancer cells [80]. Further, isorhamnetin and melatonin treatment could reduce HIF-1*α* expression via suppressing the ROS level, contributing to the inhibition of invasion and migration of human colon cancer cells [81]. L-Carnosine, an endogenously synthesized histidine dipeptide with antioxidant activity [82, 83], can mediate the generation of ATP and ROS in HCT116 cells [84]. It is reported that L-carnosine treatment decreased the HIF-1*α* protein levels and suppressed the proliferation of colon cancer cells [85]. Resveratrol, a natural phenol with antioxidant properties, reduced the expression of HIF-1*α* by scavenging ROS, leading to suppressed glucose uptake and glycolytic metabolism in colon cancer cells [86]. Overall, HIF-1*α* regulated by ROS plays a key role in gastrointestinal diseases.

## 6. HIF-1*α* Regulates ROS Levels in the GT

As an important transcriptional regulator, HIF-1*α* regulates the transcription of numerous genes involved in different cell progress [87]. HIF-1*α* can also modulate ROS generation in the GT. As has been reported, HIF-1*α* knockdown leads to increased ROS production in gastric cancer cells [74]. Consistently, there is a significant increase in ROS levels in Caco-2 treated with CoCl_2_, one of the hypoxic mimetic agents [88].

Mitochondria are the main source of intracellular ROS that oxidative phosphorylation (OXPHOS) consumes 90% to 95% of cellular oxygen, 3% of which can be transformed into superoxide [89]. Moreover, the mTOR pathway plays a key role in the regulation of HIF-1*α*. Leucine treatment can activate mTOR and subsequently activates HIF-1*α* in intestinal epithelial cells of weaned piglets; elevated HIF-1*α* inhibits OXPHOS and induces glycolysis, leading to total ROS reduction [90]. Further, HIF-1*α* knockdown increased ROS production in different gastric cancer cell lines under hypoxia [74]. In line with this, the treatment of *N*-oxalyl-D-phenylalanine, an FIH inhibitor, can ameliorate ionizing radiation-induced DNA damage and apoptosis by suppressing ROS levels [91], which indicated the effect of HIF on regulating ROS generation. However, few studies reported the underlying mechanism behind regulations of ROS by HIF-1*α* in the GT. Thus, further studies are required, which may help to develop new therapies for gastrointestinal diseases induced by ROS.

## 7. Conclusion

**Figure 3:**
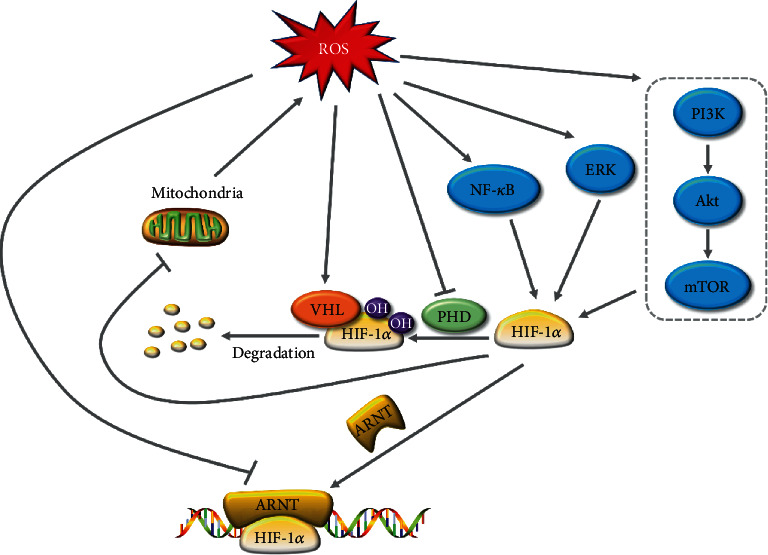
Interactions between HIF-1*α* and ROS in the gastrointestinal tract. ROS induce the expression of HIF-1*α* by activating ERK, NF-*κ*B, and PI3K/Akt/mTOR signaling pathways. Further, ROS regulate the stability and activity of HIF-1*α* by targeting VHL, PHD, and ARNT. In return, HIF-1*α* suppresses ROS generation by inhibiting mitochondrial OXHPS. HIF-1*α*: hypoxia-inducible transcription factor-1*α*; ROS: reactive oxygen species; PI3K: phosphoinositide 3-kinase; mTOR: mammalian target of rapamycin; NF-*κ*B: nuclear factor kappa-light-chain-enhancer of activated B cells; VHL: von Hippel-Lindau; PHD: prolyl 4-hydroxylase; ARNT: aryl hydrocarbon receptor nuclear translocator.

As a double-edged sword, ROS act as indispensable signaling molecules while excessive content causes oxidative tissue damage in the GT. Moreover, HIF-1*α* is also a key regulator in gastrointestinal health involved in energy metabolism, immunity, renewal, and microbial homeostasis. There are strong correlations between ROS and HIF-1*α* in the GT, which are as yet ill-defined. It is now evident that ROS regulate HIF-1*α* in different ways (Figure 3). However, the mechanism of ROS levels modulated by HIF-1*α* is vague and controversial. As the crosstalk between HIF-1*α* and ROS plays a key role in gastrointestinal diseases, it might be a promising target for disease treatment. Thus, broader and deeper studies are needed to understand the underlying mechanism of their interactions in the gastrointestinal pathologic processes.
